# Pyuria, urinary tract infection and renal outcome in patients with chronic kidney disease stage 3–5

**DOI:** 10.1038/s41598-020-76520-5

**Published:** 2020-11-10

**Authors:** I-Ching Kuo, Jia-Jung Lee, Daw-Yang Hwang, Lee-Moay Lim, Hugo You-Hsien Lin, Shang-Jyh Hwang, Hung-Chun Chen, Chi-Chih Hung

**Affiliations:** 1 Division of Nephrology, Department of Internal Medicine, Kaohsiung Medical University Hospital, Kaohsiung Medical University, 100 Tzyou First Road, Kaohsiung, 807 Taiwan; 2 Department of Internal Medicine, Kaohsiung Municipal Ta-Tung Hospital, Kaohsiung Medical University, Kaohsiung, Taiwan; 3 Faculty of Renal Care, College of Medicine, Kaohsiung Medical University, Kaohsiung, Taiwan; 4 Institute of Population Sciences, National Health Research Institutes, Miaoli, Taiwan; 5 National Institute of Cancer Research, National Health Research Institutes, Miaoli, Taiwan

**Keywords:** Nephrology, Kidney diseases

## Abstract

Pyuria is common in chronic kidney disease (CKD), which could be due to either urinary tract infection (UTI) or renal parenchymal inflammation. Only little is known regarding the association of pyuria or UTI with renal outcomes. We investigated 3226 patients with stage 3–5 CKD. Pyuria was defined as ≥ 50 WBC per high-power field (hpf) and was correlated to old age, female, diabetes, hypoalbuminemia, lower eGFR, and higher inflammation status. In Cox regression, patients with more than one episode of pyuria in the first year (11.8%) had increased risks for end-stage renal disease (ESRD) [hazard ratio (95% CI): 1.90 (1.58–2.28); *p* < *0.001*], rapid renal function progression [odds ratio (95% CI): 1.49 (1.13–1.95); *p* = *0.001*], and all-cause mortality [hazard ratio: 1.63 (1.29–2.05); *p* < *0.001*], compared to those without pyuria. In a subgroup analysis, the risk of pyuria for ESRD was modified by CKD stages. We investigated the effects of UTI (urinary symptoms and treated by antibiotics) and pyuria without UTI (urine WBC < 50 to ≥ 10/hpf without any episodes of ≥ 50 WBC/hpf or UTI), while both groups were associated with clinical outcomes. In conclusion, CKD stage 3–5 patients with frequent pyuria or UTI episodes have increased risks of renal outcomes.

## Introduction

Chronic kidney disease (CKD) is a global health problem due to its increasing incidence and prevalence worldwide^1^. To the best of our knowledge, pyuria is highly prevalent in CKD, which does not always indicate the presence of urinary tract infection (UTI)^2,3^. While pyuria is broadly defined as the appearance of leukocytes in the urine, various possible causes, including acute febrile illness, structural abnormalities of the genitourinary tract, or interstitial nephritis can also lead to sterile pyuria^4^. Previous data from a small CKD patient sample by Kwon et al. showed that the degree of pyuria was associated with UTI in patients with CKD, with the majority of their urinary WBCs being neutrophils^3^. Moreover, Hwang et al. reported that recurrent and persistent pyuria may contribute to the decline of kidney function in patients with autosomal dominant polycystic kidney disease (ADPKD)^5^.

CKD could be a risk factor for UTI, which may be present with asymptomatic bacteriuria or symptomatic UTIs requiring treatment. In addition to reduced host immunity^6^, comorbidities especially diabetes, advanced age, and urinary tract obstruction are common risks for UTI and CKD progression accordingly^7^. A short-term study of single episode UTIs^8^ and a long-term study of asymptomatic bacteriuria in diabetes revealed that there was no faster decline of renal function^9^. In fact, on the contrary, in patients with polycystic kidney disease^5,10^ and who had post-kidney transplantation^11^, UTI could be associated with renal function progression. Several factors have been proposed, particularly virulence factors such as P fimbriae on uropathogenic *E. coli*, to induce chronic inflammation^12,13^. More importantly, uremic condition in CKD alters both cellular and humoral immunity, which increases the susceptibility to a broad range of infections. However, whether pyuria or UTI has a negative impact on end-stage renal stage (ESRD) in CKD patients has never been investigated; we test this hypothesis in different definitions from an observational cohort.

## Results

### Pyuria (urine WBC ≥ 50/ hpf) in CKD patients

Out of the total of 3226 patients with stage 3–5 CKD who were not on dialysis, 1366 (42.3%) of them were female and 1448 (44.9%) had type 2 diabetes (Table 1). Pyuria (urine WBC ≥ 50/high power field [hpf]) within first year after enrollment occurred in 8.8% of males and 29.4% of females, while 11.6% non-diabetic patients and 24.8% diabetic patients had pyuria. Female and diabetic patients had more episodes of pyuria.

**Table 1:** Pyuria (Urine WBC ≥ 50/hpf) in CKD patients by sex and DM.

	All	Male	Female	*p*-value
**CKD patients without DM**				
No. of subjects	1778	1033	745	–
Pyuria within the first year				
1 episode	79 (4.4%)	19 (1.8%)	60 (8.1%)	< 0.001
> 1 episodes	128 (7.2%)	43 (4.2%)	85 (11.1%)	< 0.001
**CKD patients with DM**				
No. of subjects	1448	827	621	–
Pyuria within the first year				
1 episode	120 (8.3%)	35 (4.2%)	85 (13.7%)	< 0.001
> 1 episodes	238 (16.4%)	66 (8.0%)	172 (27.7%)	< 0.001

CKD, Chronic kidney disease; DM, Diabetes mellitus.

*P* < 0.05 indicates a significant difference between male and female.

### Characteristics and outcomes of patients by pyuria (urine WBC ≥ 50/ hpf)

In this cohort, the mean age was 63.5 ± 13.6 years, and eGFR was 24.7 ± 15.1 mL/min/1.73 m^2^, with a urine protein-to-creatinine ratio (UPCR) of 1125 (411–2553) mg/g (Table 2). Hypertension was present in 67.1% of the patients, and 26.4% of the patients had cardiovascular (CV) disease. The patients were divided into 3 groups on the basis of pyuria frequency within first year after enrollment: no pyuria episode (Group 0), 1 pyuria episode (Group 1) and > 1 pyuria episodes (Group 2) to observe the impact of frequent pyuria. The progress from pyuria Group 0 to pyuria Group 2 was associated with a gradual increase in the percentage of comorbidities, including ischemic heart disease, congestive heart failure, diabetes mellitus (DM), cancer, hypertension, and CV disease and an increase in age, C-reactive protein (CRP), phosphorus, and UPCR (Table 2).

**Table 2:** Characteristics of the CKD stage 3–5 patients divided by pyuria (Urine WBC ≥ 50/hpf) episodes.

Variable	All	Group 0	Group 1	Group 2	*p* for trend
		No pyuria	1 pyuria episode	> 1 pyuria episodes	
No. of subjects	3226	2661	199	366	
**Demographics and Medical History**					
Age (yr)	63.5 ± 13.5	62.6 ± 13.5	66.0 ± 12.9	67.9 ± 12.8	< 0.001
Gender (female)	1366 (42.3%)	964 (36.2%)	145 (72.9%)	257 (70.2%)	< 0.001
BMI (Kg/m^2^)	24.7 ± 4.0	24.8 ± 4.0	24.6 ± 4.0	24.4 ± 4.1	0.085
Mean BP (mmHg)	100.0 ± 13.8	100.0 ± 13.6	99.1 ± 13.0	100.3 ± 15.3	0.686
Smoker	354 (11.0%)	314 (11.8%)	17 (8.5%)	23 (6.3%)	0.003
Ischemic heart disease	487 (15.1%)	368 (13.8%)	40 (20.1%)	79 (21.6%)	< 0.001
CHF	413 (12.8%)	288 (10.8%)	42 (21.1%)	83 (22.7%)	< 0.001
Stroke	557 (17.3%)	405 (15.2%)	50 (25.1%)	102 (27.9%)	< 0.001
Diabetes Mellitus	1448 (44.9%)	1090 (41.0%)	120 (60.3%)	238 (65.0%)	< 0.001
Cancer	267 (8.3%)	205 (7.7%)	17 (8.5%)	45 (12.3%)	< 0.001
Hypertension	2175 (67.4%)	1754 (65.9%)	137 (68.8%)	284 (77.6%)	< 0.001
Cardiovascular disease	854 (26.5%)	637 (23.9%)	69 (34.7%)	148 (40.4%)	< 0.001
**Laboratory data**					
Hemoglobin (g/dl)	10.9 ± 2.4	11.2 ± 2.4	10.2 ± 1.9	9.7 ± 1.8	< 0.001
WBC (× 1,000 cells/μl)	7.2 ± 2.3	7.1 ± 2.2	7.6 ± 2.8	7.5 ± 2.4	< 0.001
Albumin (g/dl)	3.8 ± 0.5	3.9 ± 0.5	3.7 ± 0.5	3.6 ± 0.5	< 0.001
Cholesterol (mg/dl)	191 (162–222)	192 (164–221)	189 (159–224)	186 (155–216)	0.837
Triglyceride (mg/dl)	127 (91–185)	126 (91–184)	123 (85–179)	135 (93–187)	0.761
CRP (mg/l)	1.2 (0.4–5.3)	1.1 (0.4–5.0)	1.5 (0.5–6.4)	2.3 (0.5–9.3)	0.004
HbA1_C_ (%)	6.5 ± 1.6	6.4 ± 1.5	6.9 ± 1.9	6.8 ± 1.9	< 0.001
Potassium (mEq/l)	4.4 ± 0.6	4.4 ± 0.6	4.4 ± 0.6	4.4 ± 0.6	0.71
Phosphorus (mg/dl)	4.4 ± 1.3	4.4 ± 1.3	4.6 ± 1.2	4.8 ± 1.3	< 0.001
Calcium (mg/dl)	9.1 ± 0.8	9.1 ± 0.8	9.1 ± 0.8	9.0 ± 0.8	0.008
Bicarbonate (mEq/l)	21.7 ± 4.4	22.0 ± 4.3	21.0 ± 4.5	20.2 ± 4.5	< 0.001
Uric acid (mg/dl)	7.9 ± 2.0	7.9 ± 1.9	7.9 ± 1.9	8.0 ± 2.1	0.195
eGFR (ml/min/1.73 m^2^)	24.7 ± 15.1	26.0 ± 15.4	20.9 ± 11.9	17.9 ± 12.1	< 0.001
UPCR (mg/g)	1125 (411–2553)	1035 (370–2269)	1513 (617–3241)	2089 (765–4810)	< 0.001
**Medication**					
ACEI or ARB	1734 (53.8%)	1375 (51.7%)	128 (64.3%)	231 (63.1%)	< 0.001
Other anti-HTN drugs	1513 (45.8%)	1175 (43.4%)	103 (50.5%)	235 (60.3%)	< 0.001
Diuretics	737 (22.8%)	547 (20.6%)	66 (33.2%)	124 (33.9%)	< 0.001
OAD	972 (30.1%)	714 (26.8%)	86 (43.2%)	172 (47.0%)	< 0.001
Insulin	267 (8.1%)	182 (6.7%)	22 (10.8%)	63 (16.2%)	< 0.001
Statin	1081 (32.7%)	839 (31.0%)	83 (40.7%)	159 (40.8%)	< 0.001
**Clinical Outcomes**					
Follow-up days	1084 (651–1710)	1156 (712–1752)	1004 (581–1583)	714 (465–1246)	< 0.001
eGFR slope (ml/min/1.73 m^2^/yr)	-2.2 (-5.7 to -0.2)	-2.1 (-5.2 to -0.2)	-2.6 (-5.4 to -0.4)	-3.3 (-8.3 to -0.1)	< 0.001
Rapid eGFR decline	900 (28.1%)	699 (26.5%)	55 (27.6%)	146 (40.0%)	< 0.001
ESRD	1061 (32.9%)	819 (30.8%)	79 (39.7%)	163 (44.5%)	< 0.001
Mortality	531 (16.5%)	374 (14.1%)	44 (22.1%)	113 (30.9%)	< 0.001

CHF, congestive heart failure; BMI, body mass index; WBC, white blood cells; CRP, C-reactive protein; HbA1c, glycated hemoglobin; eGFR, estimated glomerular filtration rate; UPCR, urine protein-to-creatinine ratio; ACEI, angiotensin converting enzyme inhibitors; ARB, angiotensin receptor blocker; OAD, oral anti-diabetic drug; ESRD, end-stage renal disease.

Continuous variables are expressed as mean ± standard deviation or median (IQR), and categorical variables are expressed as number (percentage).

*P* for trend < 0.05 indicates a significant trend for increasing pyuria levels.

### Baseline parameters associated with pyuria (urine WBC ≥ 50/ hpf)

In the multivariate logistic regression analysis, older age, females, DM, CV disease, higher CRP, and higher HbA1c had a higher odds ratio (OR) for pyuria (Group 1 and Group 2) (Table 3). In contrast, higher hemoglobin, albumin, cholesterol and eGFR had a lower OR for pyuria. Glomerulonephropathy and proteinuria were not associated with pyuria, whereas tubulointerstitial nephropathy was associated with pyuria.

**Table 3:** Association between pyuria (Urine WBC ≥ 50/hpf) and parameters by multivariate logistic regression.

Variables	β	95% CI of β	*p*-value
Age (yr)	1.027	1.018 to 1.036	< 0.001
Gender (female)	4.640	3.684 to 5.845	< 0.001
BMI (Kg/m^2^)	0.987	0.962 to 1.013	0.215
MBP (mmHg)	1.007	0.999 to 1.014	0.133
Cardiovascular disease	1.358	1.083 to 1.702	0.012
Causes of CKD			
Glomerulonephropathy	1	(reference)	-
Tubulointerstitial nephropathy	1.685	1.192 to 2.382	0.003
Diabetes mellitus	1.654	1.254 to 2.181	< 0.001
Hypertention	1.023	0.693 to 1.510	0.910
Hemoglobin (g/dl)	0.925	0.860 to 0.995	0.037
Albumin (g/dl)	0.565	0.451 to 0.707	< 0.001
Log-transformed CHOL	0.173	0.068 to 0.444	< 0.001
Log-transformed CRP	1.202	1.067 to 1.353	0.002
Phosphorus (mg/dl)	0.926	0.837 to 1.023	0.131
HbA1_C_ (%)	1.134	1.058 to 1.217	< 0.001
eGFR (ml/min/1.73 m^2^)	0.986	0.975 to 0.998	0.017
Log-transformed UPCR	1.010	0.779 to 1.309	0.940

CI, confidence interval; BMI, body mass index; MBP, mean blood pressure; CHOL, total cholesterol; CRP, C-reactive protein; HbA1c, glycated hemoglobin; eGFR, estimated glomerular filtration rate; UPCR, urine protein-to-creatinine ratio.

*P* < 0.05 indicates a significantly associated with pyuria.

### Association between pyuria (urine WBC ≥ 50/ hpf) and clinical outcomes

After a median follow-up of 1084 days, 819 (30.8%), 79 (39.7%), and 163 (44.5%) of the patients developed end-stage renal disease (ESRD) in Groups 0, 1, and 2, respectively (Table 2). In the fully adjusted Cox proportional hazards model, Group 2 was associated with an increased risk for ESRD with a hazard ratio (HR) of 1.90 (95% CI, 1.58–2.28, *P* < 0.001) when compared with Group 0 (Table 4), whereas Group 1 was not associated with an increased risk.

**Table 4:** Association between pyuria (Urine WBC ≥ 50/hpf) and clinical outcomes.

	Group 0	Group 1	Group 2
	No pyuria	1 pyuria episode	> 1 pyuria episodes
**ESRD**			
Unadjusted HR	1 (reference)	1.51 (1.21–1.90)*	2.57 (2.18–3.03)*
Adjusted HR	1 (reference)	0.95 (0.75–1.21)	1.90 (1.58–2.28)*
**Rapid eGFR decline**			
Unadjusted OR	1 (reference)	1.12 (0.82–1.54)	1.92 (1.54–2.39)*
Adjusted OR	1 (reference)	1.02 (0.71–1.46)	1.49 (1.13–1.95)*
**All-cause mortality**			
Unadjusted HR	1 (reference)	1.74 (1.27–2.37)*	3.01 (2.45–3.69)*
Adjusted HR	1 (reference)	1.35 (0.98–1.86)	1.63 (1.29–2.05)*

^a^ This model was adjusted for age, sex, eGFR, log-transformed UPCR, hypertension, cardiovascular disease, diabetes, current smoker, mean blood pressure, ACE inhibitor/ARB, HbA1c, hemoglobin, albumin, BMI, log-transformed cholesterol, log-transformed CRP, and phosphorus.

* p < 0.05 compared with reference group.

The median eGFR slope was –2.2 mL/min/1.73 m^2^/year. Group 0, Group 1, and Group 2 had a rapid eGFR decline (as defined in the methods) for 699 (26.5%), 55 (27.6%), and 146 (40.0%) of the patients, respectively. In a fully adjusted multivariate logistic regression, Group 2 was significantly associated with an increased risk for rapid eGFR decline with an odds ratio of 1.49 (95% CI, 1.13–1.95, *P* = 0.001), whereas Group 1 was not associated with an increased risk (Table 4). Group 0, Group 1, and Group 2 had 374 (14.1%), 44 (22.1%), and 113 (30.9%) patients died, respectively (Table 2). In the fully adjusted Cox proportional hazards model, Group 2 was associated with an increased risk for all-cause mortality with a HR of 1.63 (95% CI, 1.29–2.05, *P* < 0.001) when compared with Group 0 (Table 4).

### Differences between UTI and pyuria without UTI (urine WBC < 50 to ≥ 10/hpf)

To clarify the effect of UTI, we divided the patients by episodes of UTI, which was less frequent than pyuria. > 1UTI episode (Group 2, n = 128) was associated with an increased risk of ESRD with a HR of 1.90 (95% CI, 1.58–2.28, *P* < 0.001) (Table 5). The associations of > 1UTI episode (Group 2) with rapid GFR decline and all-cause mortality were also significant. To clarify the effect of pyuria without UTI, we selected 2661 patients without any UTI episodes and without any high grade urine WBC (**≥ **50/hpf) and then divided them by episodes of pyuria (urine WBC < 50 to ≥ 10/hpf). > 2 episodes of pyuria without UTI (Group 2) were associated with an increased risk of ESRD with a HR of 3.53 (95% CI, 2.63–4.75, *P* < 0.001) (Table 5). > 2 episodes of pyuria without UTI (Group 2) were associated with rapid GFR decline, but not with all-cause mortality. Because the risk of pyuria without UTI for renal outcomes was numerically higher than that with UTI, we further compared the differences between baseline parameters. eGFR and UPCR were associated with pyuria without UTI, but not with UTI in multivariate logistic regression (Supplement Table 3).

**Table 5:** Association between UTI or pyuria without UTI (Urine WBC < 50 to ≥ 10/hpf) and clinical outcomes.

UTI	Group 0	Group 1	Group 2
	No UTI	1 UTI episode	> 1 UTI episodes
Number	3037	61	128
**ESRD**			
Unadjusted HR	1 (reference)	1.51 (1.21–1.90)*	2.57 (2.18–3.03)*
Adjusted HR	1 (reference)	0.95 (0.75–1.21)	1.90 (1.58–2.28)*
**Rapid eGFR decline**			
Unadjusted OR	1 (reference)	1.12 (0.82–1.54)	1.92 (1.54–2.39)*
Adjusted OR	1 (reference)	1.02 (0.71–1.46)	1.49 (1.13–1.95)*
**All-cause mortality**			
Unadjusted HR	1 (reference)	1.74 (1.27–2.37)*	3.01 (2.45–3.69)*
Adjusted HR	1 (reference)	1.35 (0.98–1.86)	1.63 (1.29–2.05)*

Pyuria without UTI	Group 0	Group 1	Group 2
(Urine WBC < 50 to ≥ 10/hpf)	No pyuria	1–2 pyuria episode	> 2 pyuria episodes
Number	2109	452	100
**ESRD**			
Unadjusted HR	1 (reference)	1.61 (1.36–1.90)*	6.42 (4.88–8.45)*
Adjusted HR	1 (reference)	1.13 (0.94–1.36)	3.53 (2.63–4.75)*
**Rapid eGFR decline**			
Unadjusted OR	1 (reference)	1.24 (0.99–1.55)	2.20 (1.46–3.31)*
Adjusted OR	1 (reference)	1.40 (1.06–1.84)*	2.01 (1.23–3.28)*
**All-cause mortality**			
Unadjusted HR	1 (reference)	1.43 (1.12–1.83)*	2.15 (1.39–3.31)*
Adjusted HR	1 (reference)	1.20 (0.92–1.56)	1.24 (0.78–1.98)

^a^The model was the same as Table 4.

*p < 0.05 compared with reference group.

### Sensitivity tests with pyuria defined as urine WBC ≥ 10/hpf

Pyuria defined as urine WBC ≥ 10/hpf within first year after enrollment was present in 18.1% of males and 29.4% of females, and it was also present in 27.1% non-diabetic patients and 43.9% diabetic patients (Supplement Table 1). The incidence of pyuria was much higher than the previously defined urine WBC ≥ 50/hpf (Table 1). The factors associated with pyuria defined by urine WBC ≥ 10/hpf were similar to those in Table 2 (data not shown). Because of the high incidence of pyuria by urine WBC ≥ 10/hpf, the patients were divided for the sensitivity test as follows: no pyuria episode (Group 0), 1–2 pyuria episodes (Group 1), and > 2 pyuria episodes (Group 2). The associations between pyuria and clinical outcomes were similar (Supplement Table 2). In the fully adjusted Cox proportional hazards model, Group 2 was associated with an increased risk for ESRD with a HR of 2.52 (95% CI, 2.10–3.01, *P* < 0.001).

### Subgroup analysis of associations between pyuria (urine WBC ≥ 50/hpf) and ESRD

**Figure 1:**
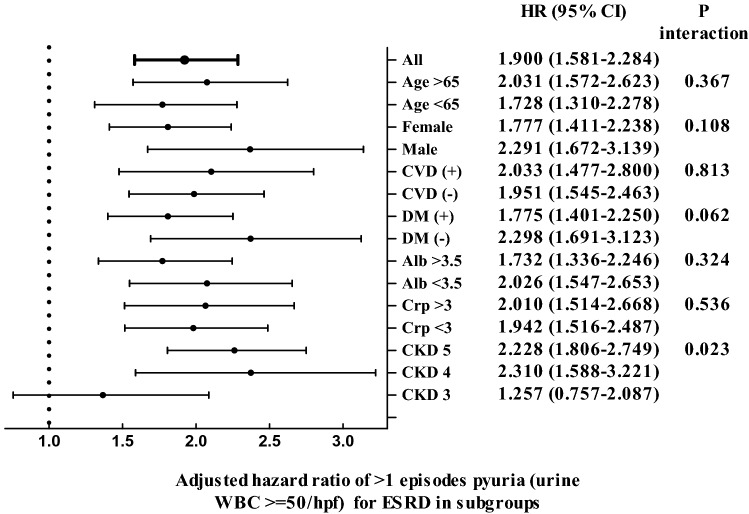
Subgroup analysis of association between > 1 episodes pyuria (urine WBC ≥ 50/hpf) and ESRD.

We also performed a subgroup analysis in groups divided according to age, gender, the presence of diabetes, CVD and CKD stages, and albumin and CRP levels (Fig. 1). The association between > 1 pyuria episode (urine WBC** ≥ **50/hpf) and ESRD was modified by CKD stages (P for interaction < 0.05). > 1 episode of pyuria was associated with a higher risk for ESRD with a HR of 2.310 (95% CI, 1.588–3.221) in CKD stage 4 and HR of 2.228 (95% CI, 1.806–2.749) in CKD stage 5 (Fig. 1), but insignificant in CKD stage 3 with a HR of 1.257 (95% CI, 0.757–2.087).

## Discussion

In this cohort of CKD patients, frequent pyuria (urine WBC ≥ 50/hpf, high probablity of nonstrile pyuria) or frequent UTI episodes, defined as > 1 episode in the first year, were associated with a higher risk for multiple relevant outcomes, including ESRD, rapid renal function progression, and all-cause mortality. However, frequent sterile pyuria (urine WBC < 50 to ≥ 10/hpf) without UTI (high probability of sterile pyuria) was significantly associated with renal outcomes. In addition, we also found that pyuria was correlated with old age, being female, diabetes, hypoalbuminemia, a lower eGFR, and a higher inflammation status in CKD patients.

Pyuria, defined as the presence of leukocytes in the urine, is present in almost every patient with symptomatic UTI and bacteriuria. The presence of pyuria could differentiate between true infection or transient colonization under the condition of asymptomatic bacteriuria. A recent study found that asymptomatic pyuria was more common in patients with CKD than in the non-CKD population and even increased with the CKD stages (24.1% in non-dialysis CKD and 51.4% in HD patients vs. 7.2% in the non-CKD population)^3^. A further analysis of 86 participants with pyuria reported that predisposing factors of UTI include the degree of pyuria, percentage of neutrophils, and presence of urinary nitrites^3^. Our study expanded the evidence to a greater CKD population, and around 17.5% of patients with stage 3–5 CKD had pyuria. Old age, being female, the presence of diabetes, hypoalbuminemia, a lower eGFR, and a higher inflammation status were related to pyuria.

Pyuria may persist in the absence of infection because of underlying inflammatory changes in the kidney, such as urinary tract stones, polycystic kidney disease, interstitial nephritis, interstitial cystitis, or systemic lupus erythematosus^2^. Since the prevalence of pyuria increased in the CKD population, it has been assumed that sterile pyuria in CKD may be due to chronic renal parenchymal inflammation. Regardless of the underlying etiology, CKD is present in a final common pathway of fibrosis as the initial insults, including nonimmune-mediated (such as diabetic nephropathy and hypertensive nephrocalcinosis) and immune-mediated (such as glomerulonephritis) kidney conditions, followed by inflammatory processes^14,15^. Under the activation of both innate and adaptive immunity, renal inflammation is characterized by inflammatory cells infiltrating in the glomeruli and tubulointerstitium, including neutrophils, macrophages, and lymphocytes^16^. Even in CKD patients with sterile pyuria, the predominant urinary WBCs were neutrophils but with a lower percentage compared with the UTI group^3^. Our study demonstrated that pyuria was associated with tubulointerstitial nephropathy but not with glomerulonephropathy. It could be supposed that tubulointerstitial injury possibly results in leakage of inflammatory cells and sterile pyuria. However, the precise correlation between the degree of cellular infiltrates and pyuria has not been elucidated. In addition, Hwang et al. found that the patients with ADPKD who had experienced recurrent and persistent pyuria in the first year had higher incidences of eGFR decline and ESRD than those without pyuria or transient pyuria^5^. The overt UTI group exhibited an even greater eGFR decline than the pyuria groups^5^. Our study showed the similar results that pyuria without UTI was associated with poor outcomes. We supposed that pyuria, especially frequent episodes, may be an indicator of chronic parenchymal inflammation, leading to poor clinical outcomes.

As for the UTIs, the clinical spectrum ranges from asymptomatic bacteriuria, to symptomatic UTIs, or to sepsis requiring hospitalization^17^. CKD could be a risk factor for UTI in addition to the elderly, female and diabetes which are common risk factors in the general population^18^. Because CKD is a chronic inflammatory state which increases susceptibility to a broad range of infections, and the comorbidities accompanied with CKD also contribute to UTI including diabetes and incomplete emptying of the urinary bladder^8^. However, there are few data available that survey for the prevalence of UTI in CKD population. In addition to virulence factors of the microorganism, an accumulation of uremic toxins impairs both cellular and humoral immunity, including the activity of phagocytosis, T lymphocytes and antigen presenting cells^19,20^. Oxidative stress and inflammatory cytokine generation in CKD also disrupt immunity^21,22^. Moreover, urinary uromodulin (Tamm-Horsfall protein) which decreases in CKD^23^, has been shown to protect against UTI by binding a type I-fimbriated bacteria^24^. These conditions may promote UTI development and determine the severity of the infection in CKD.

Although a lower amount of UTI do not promote a decline in renal function, recurrent episodes of UTI may influence the course of a pre-existing renal disease and result in kidney function deterioration. Salo et al. demonstrated recurrent childhood UTIs as a main cause of CKD as observed in patients with structural kidney abnormalities^25^. Furthermore, an acute infection of the urinary tract may lead to AKI, which is an independent risk factor for CKD development and progression^26^. Factors contributing to renal injury include direct infection of the renal parenchyma, sepsis, obstruction, vesicoureteral reflux, renal papillary necrosis, and urinary calculi^27–29^. Thakar et al. suggested that multiple episodes of AKI occur in one third of patients with AKI who survive after an initial hospitalization, and each subsequent episode doubled the risk of reaching stage 4 CKD in patients with DM^30^. As for the renal outcome of an urinary tract infection in CKD patients, the only evidence from our previous short-term study found that a single episode of UTI in a diabetic with advanced CKD possessed AKI risks and the eGFR eventually recovered^8^. This study is the first large cohort to demonstrate that multiple UTI episodes in patients with CKD resulted in renal function deterioration and increased risk of mortality. This result may imply that the severity of structural damage in the kidney accelerates the course of renal function progression when a UTI is present.

## Limitations

There are several limitations to our findings. First, the definition of clinically significant pyuria is unknown. A previous study had shown that nonsterile pyuria is correlated with the number of urine WBCs^3^. We chose urine WBC ≥ 50/hpf to study the effect of nonsterile pyuria and WBC < 50 to ≥ 10/hpf without UTI to study the effect of sterile pyuria. Second, the differentiation between pyuria with UTI and pyuria without UTI in patients with CKD is challenging. We did not perform urine culture to confirm bacteriuria in most episodes of pyuria. Asymptomatic bacteriuria could also be present in pyuria without UTI. Third, the frequency of pyuria and UTI was arbitrarily defined as the total episodes within the first year. The total measurements among patients could be different, and the frequency could be underestimated in patients reaching endpoints of less than one year. Fourth, the frequency of UTI could be underestimated due to the incomplete record from local clinics. Patients classified as pyuria without UTI might also have UTI episodes. Fifth, the causes of CKD were diagnosed clinically, not by renal biopsy.

## Conclusion

In conclusion, we found that the frequency of pyuria and UTI episodes determined the clinical outcomes in CKD stage 3–5 patients. With different definitions of pyuria, patients with more frequent episodes were at greater risk of renal outcomes independent of other major risk factors of progression. In addition, similar associations existed in patients with frequent UTI episodes. Further studies are needed to explore the effects of frequent pyuria without UTI on renal outcomes.

## Methods

### Participants and measurements

This was a prospective observational study, the Integrated CKD Care Program Kaohsiung for Delaying Dialysis, involving two affiliated hospitals of Kaohsiung Medical University in Southern Taiwan. The study was conducted from November 11, 2002 to May 31, 2009 with follow-up until May 31, 2010. We included patients who were not on renal replacement therapy and excluded patients with AKI, which we defined as more than a 50% decrease in eGFR within 3 months. Among the 3749 patients that were eligible for the study, we excluded 356 patients with stage 1–2 CKD and 77 patients with a urinary tract obstruction. After the exclusions, 3226 patients were recruited for this study. The study protocol was approved by the institutional review board of the Kaohsiung Medical University Hospital, and informed consent was obtained from all of the patients in this study. The study was carried out in accordance with relevant guidelines and regulations.

The baseline comorbidities, clinical conditions, and biochemical parameters of the patients were collected. The demographic features were recorded at the first visit, and the medical history was recorded using a chart review. DM was defined according to the World Health Organization or by the use of medication^31^. Hypertension was defined as having a systolic blood pressure (SBP) ≥ 140 mmHg, a diastolic blood pressure (DBP) ≥ 90 mmHg, or the use of antihypertensive medication. CV diseases were defined as having a clinical diagnosis of heart failure, acute or chronic ischemic heart disease, or cerebrovascular disease. Causes of CKD were classified as glomerulonephropathy, tubulointerstitial nephropathy, diabetes, hypertension and others. Glycated hemoglobin (HbA1c) was measured using an automated cation-exchange high-performance liquid chromatography. Biochemical data (hemoglobin, albumin, blood glucose, cholesterol, CRP, HbA1c, sodium, potassium, phosphorus, calcium, bicarbonate, and uric acid levels) were obtained after midnight fasting.

We performed urinalysis on patients at least every 3 months after their enrollment. Midstream urine was obtained and processed within one hour of voiding. After centrifugation, a microscopic examination was performed to determine the presence of pyuria. Urinary WBCs were counted as 0–2, 3–5, 6–10, 11–25, 26–50, 51–99, and ≥ 100 per hpf. UTI was defined as pyuria with urinary symptoms and was treated by antibiotics. We chose urine WBC ≥ 50/hpf in Tables 1, 2, 3 and 4 to study the effect of nonsterile pyuria and we chose urine WBC < 50 to ≥ 10/hpf without UTI in Table 5 to study the effect of sterile pyuria. We chose WBC ≥ 10/hpf in supplement Tables 1 and 2 to study the effect of both sterile and nonsterile pyuria. To study the impact of persistent pyuria and UTI, the frequency of pyuria and UTI was calculated within the first year after enrollment. Repeated urinalysis within 7 days after the occurrence of pyuria (urine WBC ≥ 50/hpf) was not counted as another episode.

### Outcomes

ESRD was defined as the initiation of hemodialysis, peritoneal dialysis, or renal transplantation. The initiation of renal replacement therapy was determined by reviewing the charts and the catastrophic illness cards. Kidney function was examined using the simplified modification of diet in the renal disease study equation: eGFR mL/min/1.73 m^2^ = 186 × serum creatinine^−1.154^ × age^−0.203^ × 0.742 (if the patient was female) × 1.212 (if the patient was black). The annual eGFR decline (ml/min per 1.73 m2/y) for each patient was calculated by simple linear regression with varying intercept and without covariates. A rapid eGFR decline was defined as an annual eGFR decline more than − 5 ml/min/1.73 m^2^/year. The survival status and cause of death were determined on the basis of death certificates, patients’ charts, and the National Death Index. The details of the CKD Care Program were described elsewhere^32,33^.

### Statistical analysis

The summarized statistical results of the baseline characteristics of patients were expressed as counts and percentages for the categorical data, means with standard deviation, and medians with interquartile ranges (IQR) determined for continuous variables with approximately normal distributions. A Cox proportional hazard model with a competing risk analysis was used for assessing the relationship between pyuria and renal outcomes. A multivariate logistic regression analysis was used to evaluate the relationship between pyuria and rapid renal function progression. Covariates were selected and modified from our previous studies, and continuous variables with skewed distributions were log transformed to obtain normal distributions^33^. The model was adjusted for age, sex, eGFR, log-transformed UPCR, hypertension, cardiovascular disease, diabetes, current smoker, mean blood pressure, ACE inhibitor/ARB, HbA1c, hemoglobin, albumin, BMI, log-transformed cholesterol, log-transformed CRP, and phosphorus. A *P* value of < 0.05 was considered statistically significant. Statistical analysis was performed using the R 2.15.2 software (R Foundation for Statistical Computing, Vienna, Austria) and Statistical Package for Social Sciences Version 19.0 for Windows (SPSS Inc., Chicago, IL).

## Supplementary information

Supplementary information
