# Supplementary material for: Pyuria, urinary tract infection and renal outcome in patients with chronic kidney disease stage 3–5
Source: Sci Rep. 2020 Nov 10;10:19460. doi: 10.1038/s41598-020-76520-5 (PMC7655801; doi:10.1038/s41598-020-76520-5)
Supplement: Supplementary file 1 — Supplementary information [file 41598_2020_76520_MOESM1_ESM.docx]

**Supplement Table 1. Pyuria (Urine WBC** ≥**10/hpf) in CKD patients by sex and DM**

|  | **All** | **Male** | **Female** | ***p*-value** |
| --- | --- | --- | --- | --- |
| **CKD patients without DM** |  |  |  |  |
| No. of subjects | 1778 | 1033 | 745 | - |
| Pyuria within the first year |  |  |  |  |
| 1-2 episodes | 303 (17.0%) | 94 (9.1%) | 209 (28.1%) | <0.001 |
| >2 episodes | 179 (10.1%) | 50 (4.8%) | 129 (17.3%) | <0.001 |
| **CKD patients with DM** |  |  |  |  |
| No. of subjects | 1448 | 827 | 621 | - |
| Pyuria within the first year |  |  |  |  |
| 1-2 episodes | 328 (22.7%) | 109 (13.2%) | 219 (35.3%) | <0.001 |
| >2 episodes | 307 (21.2%) | 83 (10.0%) | 224 (36.1%) | <0.001 |

UTI, Urinary tract infection; CKD, Chronic kidney disease; DM, Diabetes mellitus.

*P* < 0.05 indicates a significant difference between male and female.

**Supplement Table 2. Association between pyuria (Urine WBC** ≥**10/hpf) and clinical outcomes**

|  | **Group 0** | **Group 1** | **Group 2** |
| --- | --- | --- | --- |
|  | **No pyuria** | **1-2 pyuria**  **episode** | **>2 pyuria episodes** |
| **ESRD** | |  |  |
| Unadjusted HR | 1 (reference) | 1.57 (1.35-1.82)* | 3.59 (3.07-4.18)* |
| Adjusted HR | 1 (reference) | 1.09 (0.93-1.29) | 2.52 (2.10-3.01)* |
| **Rapid eGFR decline** |  |  |  |
| Unadjusted OR | 1 (reference) | 1.14 (0.93-1.39) | 2.12 (1.72-2.60)* |
| Adjusted OR | 1 (reference) | 1.27 (1.00-1.62) | 1.88 (1.45-2.45)* |
| **All-cause mortality** |  |  |  |
| Unadjusted HR | 1 (reference) | 1.69 (1.37-2.08)* | 2.77 (2.24-3.41)* |
| Adjusted HR | 1 (reference) | 1.38 (1.10-1.72)* | 1.63 (1.27-2.08)* |

^a^ This model was adjusted for age, sex, eGFR, log-transformed UPCR, hypertension, cardiovascular disease, current smoker, mean blood pressure, ACE inhibitor/ARB, HbA1c, hemoglobin, albumin, BMI, log-transformed cholesterol, log-transformed CRP, and phosphorus.

* p<0.05 compared with reference group

**Supplement Table 3. Association between UTI or pyuria without UTI (Urine WBC <50 to** ≥**10/hpf) and parameters by multivariate logistic regression**

|  | UTI | | |  | Pyuria withou UTI | | |
| --- | --- | --- | --- | --- | --- | --- | --- |
| variables | β | 95% CI of β | p-value |  | β | 95% CI of β | p-value |
| Age (yr) | 1.019 | 1.005 to 1.034 | 0.008 |  | 1.008 | 1.000 to 1.017 | 0.061 |
| Gender (female) | 4.546 | 3.141 to 6.580 | <0.001 |  | 6.655 | 5.224 to 8.477 | <0.001 |
| Cardiovascular disease | 1.963 | 1.408 to 2.735 | <0.001 |  | 1.139 | 0.885 to 1.464 | 0.312 |
| Cause of CKD |  |  |  |  |  |  |  |
| Glomerulonephropathy | 1 | (reference) | - |  | 1 | (reference) | - |
| Tubulointerstitial nephropathy | 1.653 | 0.929 to 2.941 | 0.087 |  | 1.379 | 0.981 to 1.937 | 0.064 |
| Diabetes mellitus | 2.117 | 1.367 to 3.280 | 0.001 |  | 1.560 | 1.162 to 2.094 | 0.003 |
| Hypertention | 0.688 | 0.333 to 1.423 | 0.313 |  | 1.265 | 0.881 to 1.816 | 0.202 |
| HbA1C (%) | 1.099 | 0.992 to 1.219 | 0.071 |  | 1.039 | 0.956 to 1.130 | 0.368 |
| eGFR (ml/min/1.73 m^2^) | 1.003 | 0.986 to 1.021 | 0.713 |  | 0.975 | 0.964 to 0.987 | <0.001 |
| Log-transformed UPCR | 0.737 | 0.495 to 1.096 | 0.132 |  | 0.761 | 0.583 to 0.993 | 0.044 |

The model is the same as the model in table 3 and also adjusted body mass index, mean blood pressure, hemoglobin, albumin,

log-transformed cholesterol, log-transformed CRP, and phosphorus.
